# Effects of grit on medical students’ wellbeing during clerkships: a longitudinal observational cohort study

**DOI:** 10.3389/fmed.2024.1331402

**Published:** 2024-05-30

**Authors:** Yung Kai Lin, Chia-Der Lin, Der-Yuan Chen, Blossom Yen-Ju Lin

**Affiliations:** ^1^Department of Surgery, Jen-Ai Hospital, Taichung, Taiwan; ^2^Department of Otorhinolaryngology Head and Neck Surgery, China Medical University Hospital, Taichung, Taiwan; ^3^School of Medicine, China Medical University, Taichung, Taiwan; ^4^Rheumatology and Immunology Center, China Medical University Hospital, Taichung, Taiwan; ^5^College of Medicine, China Medical University, Taichung, Taiwan; ^6^Department of Medical Humanities and Social Sciences, School of Medicine, Chang Gung University, Taoyuan, Taiwan

**Keywords:** perseverance, grit, workplace wellbeing, burnout, compassion satisfaction, medical student, surgical clerkship, nonsurgical clerkship

## Abstract

**Introduction:**

In medical education, the clerkship phase is a demanding period during which medical students learn to navigate the responsibilities of medical school and clinical medicine. Grit, a personal quality regarded as a non-cognitive trait, refers to perseverance and passion; specifically, it represents the ability to endure hardship and work industriously toward a goal. Most studies analysed grit as a single concept and few studies have investigated the effect of grit on the well-being of medical students through the whole-specialty training (i.e. surgical and non-surgical specialty rotations) required in clinical clerkships. Therefore, this study investigated whether associations exist between medical students’ grit, measured by the two subconstructs of perseverance and passion, and their well-being during clerkships in surgical and non-surgical specialty units.

**Methods:**

This one-year prospective web-based questionnaire study enrolled fifth-year medical students at a tertiary medical centre in central Taiwan between September 2017 and July 2018 in their first-year clerkship. The students’ sex, age, and grit were measured at the start of their clerkship. Routine surveys were conducted over one year to assess burnout and compassion satisfaction for students’ well-being, and the training specialty characteristics of the surgical and non-surgical specialty departments were recorded. This study included 92 medical students and 1,055 survey responses from individual specialty rotations. Descriptive, univariate and multivariate analyses were performed.

**Results:**

Our results revealed that medical students’ perseverance, as part of grit, was related to lower burnout and higher compassion satisfaction during clerkships, but not the subconstruct of passion. Moreover, the positive trait of perseverance measured in our study had greater explanatory power for compassion satisfaction than for burnout. Furthermore, the results revealed that older medical students suffered from less burnout than their younger counterparts, and that male medical students expressed higher compassion satisfaction than their female counterparts.

**Discussion:**

Perseverance, as a subconstruct of grit, is a positive personal quality for medical students’ clerkships, and methods driving the cultivation of perseverance in medical education should be considered. In addition, even though positive traits such as perseverance equipped medical students for compassion satisfaction, additional factors attributed to medical students’ burnout must be identified.

## Introduction

In medical education, the clerkship phase is a demanding period during which medical students learn to navigate the responsibilities of medical school and clinical medicine (1). They are provided an opportunity to assist in fulfilling the demand for patient care during clinical training (1). However, working under stressful conditions has been reported to exacerbate their burnout (2–5). It has driven the medical educators’ concerns to relieve medical students’ stress and burnout in clinical training workplaces by incorporating early and prolonged clinical skill preparation to enhance students’ confidence and technical skills (6–8) and conducting mindfulness-based interventions in clerkship (9). In addition, positive psychology examines the factors that contribute to an individual’s strength, resilience, growth, happiness, and ability to thrive in an environment that nurtures these factors (10). These factors have been emphasized and applied to promote medical students’ wellbeing during clinical training and physician-in-training in clinical workplaces (11).

Grit, a personal quality regarded as a non-cognitive trait, refers to perseverance and passion; specifically, grit represents the ability to endure hardship and work industriously toward a goal (12). The value of grit has been noted in its application in the contexts of medical education from medical students at medical schools and clinical training in clerkships, to postgraduate clinical training as resident physicians (i.e., physician-in-training). For instance, grit has been verified as a criterion for assessing the soft power of medical school applicants (13), as it counteracts burnout in medical students in their first year of medical school (14). Moreover, grit is also known to be related to higher academic performance (15, 16) and higher career interests in surgical specialties (17) among medical students. In addition, higher levels of grit were found to be a predictor of being selected for surgical training (18), receiving a grade of honor (versus high pass or pass) (19), and experiencing lower burnout in surgical clerkships (20). Previous studies also revealed the negative relationship between the grit of resident physicians and burnout, given specific specialities, such as emergency medicine residents (21) and general surgery residents (22), and as a highly valued factor for increasing the training completion rate of surgical residents (23, 24). While most studies focus on medical students’ preclinical medical school learning and clinical training in surgical clerkships and the postgraduate career stage of medical residency, studies investigating the effect of grit on the wellbeing of medical students through the whole-specialty clinical training (i.e., surgical and non-surgical specialty rotations) required in clerkships are lacking.

Moreover, the aforementioned existing studies analyzed grit as a single concept. However, grit includes two distinct personal qualities: perseverance, which refers to persistence in working toward long-term goals, and passion, which refers to “emotion compelling action” (12), or a strong desire to invest time and energy in an activity perceived as crucial (25). In a study examining high school students’ grit as a personal resource acting as a protective factor against school burnout, it was noted that only perseverance of effort was positively related to their schoolwork engagement and life satisfaction (26). Moreover, following a review of the theoretical and measurement aspects of types of grit, further exploration of the subconstructs of perseverance of effort and passion is encouraged (27).

Grit is perceived to be the main personality trait that facilitates the development of resilience in stressful circumstances (28), and medical students’ resilience has been verified as exerting a protective role on stress and quality of life in clerkship (5). Therefore, this study investigated whether associations exist between medical students’ grit, measured by the two subconstructs, namely, perseverance and passion, and their wellbeing during clerkships in surgical and non-surgical specialty units.

Our study makes significant contributions by addressing the concept of grit among medical students from a theoretical perspective (i.e., two subconstructs: perseverance and passions). Moreover, it promotes medical students’ wellbeing in the challenging clerkship period while considering the impact of different surgical and non-surgical clinical rotations on clinical training from a practical perspective.

## Methods

### Study participants

A cohort of fifth-year medical students in a six-year program at a medical school for their first-year clerkship between September 2017 and July 2018 at a tertiary medical center in central Taiwan was invited to participate. Of the 199 medical students, 151 provided written informed consent to participate (5).

### Measurement instruments

Structured and self-administered questionnaires were used in this study and consisted of the following measures.

#### Grit

The grit of medical students was measured using the Short Grit Scale (29). The perseverance and passion subcategories were measured using four items each, all of which were scored on a five-point scale from 1 (*not at all like me*) to 5 (*very much like me*). We reversed the coding scores for passion in the analysis due to the original negative wording. A factor analysis was implemented to validate the items for perseverance and passion, and the results revealed identical loading patterns for their factors (29). The Cronbach’s α values of perseverance and passion were 0.784 and 0.589, respectively. The detailed statistical results are provided in Appendix 1. Factor scores for perseverance and passion were calculated using regression analysis.

#### Workplace wellbeing at individual clinical specialty rotation follow-up

The question items for measuring burnout and compassion satisfaction as factors of medical students’ wellbeing were adapted from the Professional Quality of Life Scale, Version 5. Burnout refers to negative emotions associated with perceived difficulties and feelings of hopelessness in managing work tasks, while compassion satisfaction refers to pleasure derived from helping people (30). Scoring was based on a five-point Likert-type scale: 1 (*never*), 2 (*seldom*), 3 (*sometimes*), 4 (*often*), and 5 (*always*) (31). Individual factor analyses were implemented to validate the burnout and compassion satisfaction results. The Cronbach’s α values for burnout and compassion satisfaction were 0.856 and 0.960, respectively. Means were calculated to evaluate the extent of burnout and satisfaction with compassion; detailed information is provided in Appendix 2.

#### Other measures: medical students’ demographics and rotating specialty characteristics

Medical students’ sex and age were included as confounding factors for grit in training contexts for their wellbeing during clinical training (23). Furthermore, medical students’ rotating specialties were recorded and categorized as surgical and non-surgical specialties as control variables for their wellbeing during clinical training. Surgical specialties included breast, colorectal, cardiovascular, critical, general, pediatric, and thoracic surgery, while the non-surgical specialties were cardiology, chest and intensive care, gastroenterology, general medicine, hematology and oncology, infectious diseases, metabolism, nephrology, neurology, obstetrics and gynecology, pediatrics, psychiatry, radiology, and rheumatology. Some specialties were required for all medical students in clerkship training, whereas others were elective.

### Study procedures

This study was approved by the Research Ethics Committee of the China Medical University and Hospital, Taichung, Taiwan [CRREC-106-067; CRREC-106-067 (AR-1)].

A baseline survey was conducted to assess students’ personal backgrounds and qualities at the beginning of their clerkships, and follow-up surveys were conducted to assess students’ training wellbeing during each clinical specialty rotation. Since this study longitudinally traced medical students’ training for approximately 1 year, they voluntarily completed individual follow-up surveys based on individual specialty rotations. This study included medical students who completed the baseline and follow-up clinical specialty rotation surveys at least three times over the course of their one-year clerkship (32), which provided the required data on medical students’ age, sex, grit, and training wellbeing during each clinical specialty rotation. The study included 92 medical students, comprising 44 men (48%) and 48 women (52%), with an average age of 23 years [mean = 22.891, standard deviation (SD) = 0.926]. The average grit component scores were 3.433 (SD = 0.693) for perseverance and 3.177 (SD = 0.613) for passion. Participants’ personal background information is presented in Table 1.

**Table 1:** Demographic characteristics of participating medical students (*n* = 92).

Variable	Freq.	%	Mean	SD
Demographics				
Sex				
Male	44	48		
Female	48	52		
Age (year)			22.891	0.926
Personal quality: grit^†^				
Perseverance			3.433	0.693
Passions			3.177	0.613

^†^Grit was measured using the Short Grit Scale developed by Duckworth and Quinn (19) and by assessing the two subcategories of perseverance and passion. Please refer to Appendix 1 which presents the descriptive statistics and statistics of the construct validity and reliability of grit.

### Statistical analyses

We employed descriptive analyses to examine medical students’ demographic characteristics, grit levels (i.e., perseverance and passion levels), and perceived training wellbeing (i.e., burnout and compassion satisfaction) with respect to individual specialty rotations during their clerkships. One might argue that the data in this study could be analyzed using a two-level or multilevel analysis due to medical students’ repeated responses regarding their burnout and compassion satisfaction longitudinally with respect to their demographics, personal quality, and contexts. Since the intraclass correlation coefficients for medical students’ burnout and compassion satisfaction (0.003 and 0.004, respectively) in individual specialty rotations were smaller than 0.05, the minimum cut-off point for performing multilevel analysis (33, 34), multilevel effects were ignored in this dataset.

With medical students’ wellbeing measured as the means of burnout and compassion satisfaction as dependent variables, univariate analyses were performed using simple regressions and multiple regression analyses were further performed. Statistical analyses were performed using SPSS software (version 25.0; IBM, Armonk, NY, United States).

## Results

Overall, the study included 92 medical students who provided 1,055 responses to the clinical specialty rotation surveys, with an average of 12 responses on the rotation surveys per medical student (minimum = 3, maximum = 19).

Analysis of 1, 055 survey responses from medical students revealed 207 responses for surgical training (20%) and 848 for non-surgical training (80%) clerkships. The average perceived burnout score was 2.347 (SD = 0.617), and the average compassion satisfaction score was 3.504 (SD = 0.819) during clerkships across all specialty rotations. Furthermore, the average score was 2.419 (SD = 0.627) for burnout and 3.444 (SD = 0.845) for compassion satisfaction in surgical specialty training, while the average score was 2.329 (SD = 0.613) for burnout and 3.519 (SD = 0.813) for compassion satisfaction in non-surgical specialty training. However, no statistically significant differences were found in medical students’ burnout or compassion satisfaction between surgical and non-surgical specialties. Detailed results of the descriptive analyses of medical students’ burnout and compassion satisfaction during individual specialty rotations are presented in Table 2.

**Table 2:** Medical students’ wellbeing during clinical specialty rotations (*n* = 1,055).

Variable	Total (*n* = 1,055)^†^		Surgical specialty training (n1 = 207)		Non-surgical specialty training (n2 = 848)	
	Mean	SD	Mean	SD	Mean	SD
Wellbeing						
Burnout	2.347	0.617	2.419	0.627	2.329	0.613
Compassion Satisfaction	3.504	0.819	3.444	0.845	3.519	0.813

Burnout and compassion satisfaction were measured using the Professional Quality of Life Scale, Version 5, with 10 items each (20, 21). ^†^Please refer to Supplementary Appendix 2 for descriptive statistics and statistics of the construct validity and reliability of medical students’ wellbeing. No statistically significant difference was found in medical students’ burnout or their compassion satisfaction in surgical or non-surgical specialty units by individual *t*-tests.

Table 3 shows the univariate analyses of the predictors of burnout and compassion satisfaction among medical students. It reveals that the medical students’ perseverance (*β* = −0.335, *p* < 0.001), passion (*β* = −0.071, *p* < 0.05), and older age (*β* = −0.096, *p* < 0.01) were related to reduced burnout. On the other hand, the medical students’ perseverance (*β* = 0.451, *p* < 0.001) and male sex (*β* = −0.205, *p* < 0.001) were related to increased compassion satisfaction. However, there was no difference in perceptions of burnout or compassion satisfaction between medical students undergoing surgical and non-surgical training (*p* > 0.05).

**Table 3:** Univariate analyses of determinants of medical students’ wellbeing (*n* = 1,055).

Variables	Burnout		Compassion satisfaction	
	Std. β	95% CI	Std. β	95% CI
Perseverance	−0.335***	−0.242, −0.172	0.451***	0.326, 0.414
Passions	−0.071*	−0.081, −0.007	−0.047	−0.088, 0.011
Sex (default = male)	0.053	−0.010, 0.141	−0.205***	−0.437, −0.241
Age	−0.096**	−0.098, −0.022	0.023	−0.031, 0.070
Rotating specialty type (default = non-surgical specialty)	0.057	−0.004, 0.183	−0.037	−0.200, 0.049

*p < 0.05; **p < 0.01; ***p < 0.001.

The multiple regression analyses (Table 4) revealed a direct negative association between medical students’ perseverance in grit and burnout (*β* = −0.332, *p* < 0.001) and a direct positive association between their perseverance in grit and compassion satisfaction (*β* = 0.428, *p* < 0.001); passion in grit did not show the same results (*p* > 0.05). Moreover, the positive trait of perseverance measured in our study has a larger explanatory power for compassion satisfaction (partial correlation = 0.430) than that for burnout (partial correlation = −0.330). Older medical students experienced less burnout than younger medical students (*β* = −0.078, *p* < 0.01), and male medical students expressed higher compassion satisfaction than their female counterparts (*β* = −0.125, *p* < 0.001). Furthermore, the results indicated no difference in the perceptions of burnout or compassion satisfaction between medical students undergoing surgical and non-surgical training (*p* > 0.05).

**Table 4:** Multiple regressions of the determinants of medical students’ wellbeing (*n* = 1,055).

Variables	Burnout			Compassion satisfaction		
	Std. β	Partial corr.	95% CI	Std. β	Partial corr.	95% CI
Perseverance	−0.332***	−0.330	−0.240, −0.169	0.428***	0.430	0.306, 0.395
Passions	−0.055	−0.057	−0.070, 0.002	−0.032	−0.035	−0.072, 0.019
Control variables						
Sex (default = male)	0.002	0.002	−0.071, 0.075	−0.125***	−0.136	−0.299, −0.115
Age	−0.078**	−0.081	−0.086, −0.013	0.019	0.021	−0.030, 0.062
Rotating specialty type (default = non-surgical specialty)	0.053	0.056	−0.006, 0.171	−0.036	−0.040	−0.184, 0.037
R2	0.126			0.222		

Burnout and compassion satisfaction were measured as dependent variables in multiple regressions. To avoid multicollinearity, the perseverance and passion values were calculated as factor scores using factor analysis. **p < 0.01; ***p < 0.001.

## Discussion

Using a cohort of fifth-year medical students in a six-year program at a medical school for their first-year clerkship as study subjects, this study demonstrated the value of medical students’ perseverance of grit during their first-year clerkships by tracking their burnout and compassion satisfaction for 1 year. The results also revealed that older medical students experienced less burnout than their younger counterparts and that male medical students expressed higher compassion satisfaction than their female counterparts.

This study revealed that, as a subconstruct of grit, medical students’ perseverance functioned as a critical personal resource during their clinical training in clerkships, directly related to lower burnout and higher compassion satisfaction during clerkship training (Table 4). Perseverance refers to the belief that diligent and consistent work produces positive results and relates to the process by which individuals achieve their goals (35). Medical students must refine their practical skills in clinical training, which is more demanding than the acquisition of theoretical knowledge that is required in preclinical academic life (16). Thus, faced with such demands, medical students may employ perseverance as a coping mechanism to overcome challenges during clinical training (36).

However, this study revealed that the other subconstruct of grit, medical students’ passion, was not associated with their clinical training wellbeing, such as burnout and compassion satisfaction in clerkships. Previous studies have argued that students’ passion, as a remedy for work-related discouragement (37), may enhance their emotional strength, allowing them to summon the energy and enthusiasm (36) required to face challenges. However, Jachimowicz et al. (38) defined passion of grit as “a strong feeling toward a personally important value/preference that motivates intentions and behaviors to express that value/preference” and hence, passion must be connected to purpose (39). However, in our study, the question items for passion in grit measured a general emotion rather than medical aspiration. We speculate that the measure of passion in grit in general may not fully reflect medical students’ values and preferences in workplace-based training; therefore, we were unable to verify the value of medical students’ passion in grit as a direct effect on their wellbeing.

In addition to the current investigation of perseverance of grit as a valuable personal resource for medical students, other studies have identified medical students’ traits and explored how they can be used to predict their performance in academic fields and clinical training. For example, medical students’ core self-evaluation tendencies positively affected workplace compassion satisfaction and burnout during clerkships (32). Furthermore, more positive preclinical nontechnical attributes (i.e., higher preclinical moral, social, and physical performance) predicted a lower level of burnout in medical students during clerkships (5). Emotional stability and openness—as defined by the Big 5 Personality Test—were also related to lower burnout in students during their clerkship years (40). Further, non-cognitive capacities were recently investigated to determine their predictive validity for learners, educators, and institutions, and thereby broaden the value of their application (41). This study’s findings regarding perseverance could be valuable in this perspective.

It deserves to be noted further that grit covering the two subconstructs of perseverance and passion can be nurtured over time from adolescence (42) to continued academic study (43, 44) and work-based learning (42). The education curriculum on the recognition and management of stress has been verified to increase medical trainees’ perseverance subscales of grit in clerkships (45). Furthermore, several potential strategies exist for healthcare organizations to become clinical education institutions to grit development, such as an emphasis on inclusivity, young trainee engagement, and mentorship (46). Further longitudinal studies of medical students’ to physicians’ perseverance or grit and wellbeing are required to better understand how these personal qualities might change and be related to medical professionals’ careers in medicine. Moreover, it should be noted that the positive trait of perseverance measured in our study has a larger explanatory power for compassion satisfaction than that for burnout (Table 4). We argue that even for positive traits such as perseverance, equipped for medical students’ compassion satisfaction, additional contextual demands attributed to medical students’ burnout must be identified.

Our results also revealed that older medical students experienced less burnout during clerkships than their younger counterparts. Medical students’ age was verified to be a critical predictor of burnout (47). Some evidence has indicated that junior doctors are often unaware of how to access help and support (48). Moreover, factors in the work environment such as perceived stigma, negative personal experiences, and the hidden curriculum may contribute to the tendency of medical students with burnout to not seek help (49). Notably, it was argued that young medical students had a high risk of psychological problems during the COVID-19 pandemic (50). Therefore, clinical clerkships should create a supportive work environment for medical students and address individual practices and attitudes that foster resilience (48, 51).

The study results demonstrated that female medical students expressed less compassion satisfaction during clerkships than their male counterparts. Compassion satisfaction was defined as the pleasure derived by medical students from doing their work well and thereby helping people (34). Some studies have revealed that women study more diligently than do men to satisfy gender role expectations within medicine (52, 53). A series of experiments also indicated that women were more likely to underestimate their ability than were men when completing a stereotypically male task related to mathematics and science (54). Furthermore, female students reported that they received less feedback or encouragement from their supervisors during clinical training than male students did, which may have negatively influenced the sense to which they felt prepared and clinically capable (55). Although our study did not further analyze this finding, we recognize that clinical supervisors must address concerns related to sex and gender during medical students’ clinical clerkships, such as workload, relaxation, social adaptation, and personal and financial matters; investigating these factors may indicate the reasons for our findings (56).

This study did not find any differences in perceived burnout or compassion satisfaction between students undergoing surgical and non-surgical training. Furthermore, although grit has been explored against the backdrop of medical students’ surgical training (18–20), we argue that perseverance, as a factor of grit, could also be applied to non-surgical specialty training and prove to be beneficial to medical students’ wellbeing, given all the rotating specialties included in this study. Moreover, we suggest the continued observation of new cohorts to ensure the wellbeing of medical students undergoing clerkships, given the negative effects of the COVID-19 pandemic on surgical and non-surgical clinical training; for example, students have likely been forced to engage in remote learning with limited on-site participation (57, 58).

Despite its clear contributions, this study had several limitations. First, the self-reported data employed to examine personal traits, such as grit, may have introduced social desirability or self-serving biases (59). However, the use of reports by faculty members would also be problematic; supervisors may be unfamiliar with trainees and thus overestimate their grit level due to a lack of interaction and insufficient observation time (60). Thus, to reduce self-reporting bias, we avoided the explicit mention of grit in the questionnaire headings to prevent the respondents’ answers from being influenced. Second, we employed a baseline survey conducted at the beginning of the students’ clerkships to determine their grit levels, and tracked the students during their clinical training over a one-year period. However, grit can vary over time; the same is true for its subcategories of perseverance and passion (61). Third, this longitudinal observational cohort study of medical students from a single medical school had a small sample size (*n* = 92) and a response rate of 46% (92/199); therefore, the results may not be nationally representative. The sample size in our study was smaller than that suggested by the National Education Association at 132 for a population of 199 medical students in one cohort (62). However, our longitudinal data (1,055 responses) exceeded the required data amount with respect to the observed variables (31 variables in this study) based on the standards for multivariate statistics in the social sciences (63, 64). Furthermore, future studies should analyze how medical students employ perseverance during unusual events, such as the COVID-19 pandemic.

## Conclusion

Our study results revealed that perseverance, as a subconstruct of grit, is a positive personal quality for medical students’ clerkships, and methods driving the cultivation of perseverance in medical education should be considered. Moreover, the positive trait of perseverance measured in our study had a larger explanatory power for compassion satisfaction than for burnout among medical students during clinical clerkships. Therefore, additional factors that contribute to medical student burnout must be identified.

## Data availability statement

The datasets presented in this article are not readily available because the participant identity might be recognized given the information of certain contexts including medical facility and data collection year in this study. Requests to access the datasets should be directed to the corresponding author upon reasonable request and discussion.

## Ethics statement

The studies involving humans were approved by the Research Ethics Committee, China Medical University and Hospital, Taichung, Taiwan, ROC [CRREC-106-067; CRREC-106-067 (AR-1)]. The studies were conducted in accordance with the local legislation and institutional requirements. The participants provided their written informed consent to participate in this study.

## Author contributions

YKL: Conceptualization, Formal analysis, Funding acquisition, Investigation, Methodology, Writing – original draft, Writing – review & editing. C-DL: Conceptualization, Funding acquisition, Investigation, Writing – review & editing. D-YC: Conceptualization, Investigation, Writing – review & editing. BY-JL: Conceptualization, Data curation, Formal analysis, Funding acquisition, Investigation, Methodology, Project administration, Resources, Software, Supervision, Validation, Visualization, Writing – original draft, Writing – review & editing.
