# Supplementary material for: Effects of grit on medical students’ wellbeing during clerkships: a longitudinal observational cohort study
Source: Front Med (Lausanne). 2024 May 30;11:1331402. doi: 10.3389/fmed.2024.1331402 (PMC11169821; doi:10.3389/fmed.2024.1331402)
Supplement: Supplementary file 1 [file Presentation_1.pdf]

Appendix 1. Descriptive analyses and construct validity and reliability for medical students' grit (n = 92)

Variable	Mean (Freq.)	SD (%)	Factor loadings	Cronbach's α value
**Grit† (scale: 1 to 5)**				
Perseverance	**3.433**	**0.693**		**0.784**
I am diligent	3.165	0.834	0.768	
I am a hard worker	3.598	0.984	0.711	
I finish whatever I begin	3.380	0.936	0.682	
Setbacks do not discourage me	3.576	0.842	0.679	
Passions	**3.177**	**0.613**		**0.589**
I often set a goal but later choose to pursue a different one (R)	3.391	0.937	0.585	
I have difficulty maintaining my focus on projects that take more than a few months to complete (R)	3.424	0.975	0.550	
I have been obsessed with a certain idea or project for a short time but later lost interest (R)	3.076	0.940	0.443	
New ideas and projects sometimes distract me from previous ones (R)	2.815	0.797	0.429	

Notes: Grit was measured using the Short Grit Scale developed by Duckworth and Quinn[19] and by assessing the two subcategories of perseverance and passion with four items each. (R) refers to reverse coding of the original scores (1→5, 2→4, 4→2, 5→1).
