# Supplementary material for: Effects of grit on medical students’ wellbeing during clerkships: a longitudinal observational cohort study
Source: Front Med (Lausanne). 2024 May 30;11:1331402. doi: 10.3389/fmed.2024.1331402 (PMC11169821; doi:10.3389/fmed.2024.1331402)
Supplement: Supplementary file 2 [file Presentation_2.pdf]

Appendix 2. Descriptive analyses and factor analyses for medical students' well-being during clinical specialty rotations (*n* = 1,055)

Variable	Mean	SD	Factor loadings	Cronbach's α value
**Burnout (scale: 1 to 5)**	**2.347**	**0.617**		**0.856**
I am happy (R)	2.323	0.762	0.732	
I feel connected to others (R)	2.174	0.846	0.647	
I am not as productive at work because I am losing sleep over traumatic experiences of a person I help	1.913	0.831	0.479	
I feel trapped by my job as a medical doctor	2.310	0.932	0.759	
I have beliefs that sustain me (R)	2.333	0.973	0.793	
I am the person I always wanted to be (R)	2.188	0.986	0.780	
I feel worn out because of my work as a medical doctor	2.677	1.037	0.553	
I feel overwhelmed because my work load seems endless	2.261	0.934	0.673	
I feel "bogged down" by the system	2.676	1.032	0.511	
I am a very caring person (R)	2.613	0.993	0.664	
**Compassion Satisfaction (scale: 1 to 5)**	**3.504**	**0.819**		**0.960**
My work makes me feel satisfied	3.559	0.882	0.856	
I feel invigorated after working with those I help	3.661	0.955	0.856	
I like my work as a medical doctor	3.635	0.950	0.880	
I am pleased with how I am able to keep up with helping techniques and protocols	3.545	0.995	0.864	
I get satisfaction from being able to help people	3.597	0.952	0.888	
I have happy thoughts and feelings about those I help and how I could help them	3.749	0.932	0.878	
I believe I can make a difference through my work	3.516	0.973	0.874	
I am proud of what I can do to help	3.454	0.993	0.902	
I have thoughts that I am a "success" as a medical doctor	2.739	0.964	0.677	
I am happy that I chose to do this work	3.576	0.963	0.884	

Note: Medical students' burnout and compassion satisfaction were measured using the Professional Quality of Life Scale, Version 5, with 10 items each.[20,21] (R) refers to reverse coding of the original scores (1→5, 2→4, 4→2, 5→1).
